# Recombinant Tropomyosin from the Pacific Oyster (*Crassostrea gigas*) for Better Diagnosis

**DOI:** 10.3390/foods11030404

**Published:** 2022-01-30

**Authors:** Roni Nugraha, Thimo Ruethers, Aya C. Taki, Elecia B. Johnston, Shaymaviswanathan Karnaneedi, Sandip D. Kamath, Andreas L. Lopata

**Affiliations:** 1 Department of Aquatic Product Technology, Faculty of Fisheries and Marine Science, IPB University, Bogor 16680, Indonesia; rnugraha@apps.ipb.ac.id; 2 Molecular Allergy Research Laboratory, College of Public Health, Medical and Veterinary Sciences, James Cook University, Douglas 4811, Australia; thimo.ruethers@my.jcu.edu.au (T.R.); aya.taki@unimelb.edu.au (A.C.T.); elecia.johnston@jcu.edu.au (E.B.J.); shaymaviswanathan.karnaneedi@my.jcu.edu.au (S.K.); sandip.kamath@jcu.edu.au (S.D.K.); 3 Australian Institute of Tropical Health and Medicine, James Cook University, Douglas 4811, Australia; 4 Centre for Sustainable Tropical Fisheries and Aquaculture, Faculty of Science and Engineering, James Cook University, Douglas 4811, Australia; 5 Centre for Food and Allergy Research, Murdoch Children’s Research Institute, Parkville 3052, Australia; 6 Tropical Futures Institute, James Cook University Singapore, Singapore 387380, Singapore; 7 Department of Veterinary Biosciences, Melbourne Veterinary School, The University of Melbourne, Melbourne 3010, Australia

**Keywords:** Cra g 1, molecular allergology, tropomyosin, Pacific oyster, mollusc allergen, shellfish allergy, food allergy, recombinant allergen

## Abstract

The Pacific oyster is a commercially important mollusc and, in contrast to most other shellfish species, frequently consumed without prior heat treatment. Oysters are rich in many nutrients but can also cause food allergy. Knowledge of their allergens and cross-reactivity remains very limited. These limitations make an optimal diagnosis of oyster allergy difficult, in particular to the Pacific oyster (*Crassostrea gigas*), the most cultivated and consumed oyster species worldwide. This study aimed to characterise IgE sensitisation profiles of 21 oyster-sensitised patients to raw and heated Pacific oyster extract using immunoblotting and advanced mass spectrometry, and to assess the relevance of recombinant oyster allergen for improved diagnosis. Tropomyosin was identified as the major allergen recognised by IgE from 18 of 21 oyster-sensitised patients and has been registered with the WHO/IUIS as the first oyster allergen (Cra g 1). The IgE-binding capacity of oyster-sensitised patients’ IgE to purified natural and recombinant tropomyosin from oyster, prawn, and dust mite was compared using enzyme-linked immunosorbent assay. The degree of IgE binding varied between patients, indicating partial cross-sensitisation and/or co-sensitisation. Amino acid sequence alignment of tropomyosin from these three species revealed five regions that contain predicted IgE-binding epitopes, which are most likely responsible for this cross-reactivity. This study fully biochemically characterises the first and major oyster allergen Cra g 1 and demonstrates that the corresponding recombinant tropomyosin should be implemented in improved component-resolved diagnostics and guide future immunotherapy.

## 1. Introduction

Consumption of shellfish has increased considerably in the last two decades due to the high nutrient content and health benefits [1]. Two large invertebrate groups, crustacean and mollusc, form the shellfish group and consist of hundreds of different species consumed globally. The Food and Agricultural Organization lists over 60 crustacean and 120 mollusc species that are widely consumed (FAO http://www.fao.org/fishery/statistics/global-consumption/en (accessed on 6 September 2017). However, the increasing consumption of shellfish has been associated with the increasing incidence of allergic reactions to shellfish. Many studies have shown that dietary habits correlate directly with food allergy prevalence. In Asia, for example, the prevalence of seafood allergy is much higher as compared to Australia, where milk and peanut allergies are more prevalent [2,3,4]. Furthermore, many consumers are introduced to new shellfish species due to the better distribution and globalisation of shellfish-based food products. 

The Pacific oyster (*Crassostrea gigas*) is one of the most important edible molluscs in the world, collected from the wild and increasingly grown in aquaculture. However, the knowledge of their allergens and possible allergenic cross-reactivity remains very limited [5,6,7]. In previous studies [6,8], a serum pool from shellfish allergic patients demonstrated immunological reactivity to a 38 kDa protein band. Subsequent analysis using mass spectrometry identified tropomyosin (TM) as the major allergenic protein component. TM was also identified by our group as the major allergen in the Sydney rock oyster (*Saccostrea glomerata*) [9]. Tropomyosin (TM), a structural protein involved in muscle contraction, causes allergic sensitization and IgE antibody reactivity in up to 80% of shellfish allergic patients and is found in most shellfish species that have been studied so far [10]. Due to the high similarity in amino acid sequence among TM from shellfish and other invertebrate species (e.g., house dust mites), sensitised individuals often experience allergic reactions to multiple species. However, many clinical studies have demonstrated that the serum IgE antibody repertoire differs in allergic patients [10,11]. Various factors, including stability of the allergen and the route of sensitisation e.g., ingestion or inhalation, could affect the IgE reactivity. 

Several studies have demonstrated that the use of purified allergen components can better predict clinical allergy reactivity compared to SPT and/or specific-IgE to whole protein extract [12,13,14]. Currently, however, no purified allergen components are available for the diagnosis of oyster allergy [5]. In addition, the species used for diagnostics are native to the Northern hemisphere, and several studies have demonstrated that allergen components often differ between species of the Northern and Southern hemispheres [15,16], posing a challenge for reliable diagnostics. Immunological studies of natural and recombinant oyster TM have been reported previously [17,18,19]. However, these studies did not register this major allergen with the World Health Organization/International Union of Immunological Societies (WHO/IUIS) as only a very limited number of patients were evaluated, and the reactivity to other TM was determined using IgE derived from pooled rather than individual serum. Therefore, in this study, TM from the Pacific oyster was purified and expressed as a recombinant protein, and its structural properties and IgE reactivity were characterised to evaluate its utility for improved component-resolved diagnostics. Cross-species IgE-reactivity with one commonly consumed crustacean species, the black tiger prawn (*Penaeus monodon*), and major inhalant allergen source, house dust mite, was also determined.

## 2. Materials and Methods

### 2.1. Patient Selection

Twenty-one subjects with a clinical history of IgE-mediated allergic reaction(s) to shellfish and positive oyster-specific IgE by ImmunoCAP (>0.35 kUA L–1; Phadia Pty Ltd., Uppsala, Sweden) were recruited at The Alfred Hospital Allergy Clinic, Melbourne, VIC, Australia (see Table 1 for patient characteristics). Oral challenges with molluscs were not conducted with these patients, in accordance with the clinicians’ preference for safer serum-specific allergen IgE testing due to comorbidities and clinical history of reactions on exposure. 

### 2.2. Sample Preparation and Protein Extraction

Raw and heated oyster extracts were prepared according to the method described in detail in a previous study [8]. Briefly, five grams of minced fresh Pacific oysters were homogenized in 25 mL of TBSN buffer (25 mM Tris; 3.0 mM KCl; 1 M NaCl, pH 7.4) and stirred at 4 °C overnight. The extract was centrifuged at 15,000× *g* for 15 min at 4 °C and the obtained supernatant was further filtered through a 0.45-µm membrane to attain the final raw extracts. To obtain the heated extracts, an aliquot of the raw extract was heated at 95–100 °C in a water bath for 15 min.

### 2.3. Quantification of Total Protein

Total protein from the raw and heated extract was determined using the BCA Assay (Thermo Scientific, Waltham, MA, USA) following the manufacturer’s instruction. A pre-diluted set of bovine serum albumin (Pierce, Waltham, MA, USA) was used as protein standards.

### 2.4. Purification of Natural TM from the Pacific Oyster

Natural oyster TM was purified through ion-exchange chromatography using Biologic LP FPLC (BioRad, Hercules, CA, USA). Hence, 20 mg of protein was diluted in buffer containing 5 mM NaPO_4_, 150 mM NaCl, pH 6.8, and loaded onto a Bio-scale Mini CHT Ceramic Hydroxyapatite Cartridges column (BioRad, Hercules, CA, USA). An increasing concentration of phosphate was used to elute the proteins and purified proteins were stored at −20 °C until further use. 

### 2.5. Cloning, Sequencing and Expression of Recombinant Oyster TM

Total RNA from oyster muscle tissue was isolated using TriZol reagent (Life Technologies, Molecular, Australia) following the manufacturer’s instruction. Using a cDNA Synthesis kit (Bioline, Eveleigh, Australia) single-stranded cDNA were generated from RNA. Forward (5′ CGC AGA ATT CAT GAC AGC ATC AAG AAG AAG ATG 3′) and reverse primer (5′ CGA ACC TGC AGT TAA TAT CCT GCC AGC TCG G 3′) derived from the nucleotide sequence for oyster TM (GenBank ID AB444943.1) were used to amplify the TM region from the generated cDNA. Using the TOPO TA cloning kit (Invitrogen, Waltham, MA, USA), the PCR products were cloned into the sequencing vector, pCR 2.1, and transformed into chemical competent *Escherichia coli* (TOP10). Blue-white screening and colony PCR were used to confirm the positive colonies. The plasmid containing the TM open reading frame was sequenced by Macrogen Inc., Seoul, Korea. 

For the expression of recombinant TM, the coding region of the protein was subcloned into pRSET-A bacterial expression vectors. The cloned vectors were transformed into BL21 (DE3) RIPL *E. coli* competent cells. The cells were grown on a lysogeny broth (LB) agar plate with 0.1 mg/mL of ampicillin at 37 °C overnight. A single colony was selected and further grown in 10 mL of LB media overnight. TM was expressed by adding 1 mL of overnight LB media to the Novagen^®^ Overnight Express™ Autoinduction Systems (Merck, Kenilworth, NJ, USA). After 24 h, cells were harvested and lysed using a probe sonicator. Expressed TM was purified using HisPur™ Ni-NTA column (Thermo Scientific, Waltham, MA, USA).

### 2.6. SDS-PAGE and Immunoblotting Using Patient IgE-Antibody

The oyster extracts and purified TM were resolved using AnykD™ Criterion™ TGX™ Precast Midi Protein Gel (BioRad, Hercules, CA, USA). A solution of protein containing 10 µg of protein or 2 µg of purified TM was added to each well and separated on an electrophoresis apparatus at 170 V for 1 h. The gel was stained with Coomassie Brilliant Blue using the protocol described in a previous study [6].

To analyse serum IgE binding, 100 µg of extracts or 20 µg of purified proteins were resolved using AnykD™ Criterion™ TGX™ Precast Midi Protein Gel (BioRad, Hercules, CA, USA). Using the Trans-Blot^®^ SD Semi-Dry Electrophoretic Transfer Cell (BioRad, Hercules, CA, USA), the separated proteins were transferred to a nitrocellulose membrane and the membrane was blocked with 0.1% casein blocking solution (Sigma, St. Louis, MO, USA) for 1 h. 

The membrane was washed three times using PBS supplemented with 0.05% Tween (PBST). The blocked-nitrocellulose membrane was incubated overnight with individual serum samples diluted 1:20 in PBST added with 0.2× casein. After washing, anti-human IgE (1:10,000 dilution, Dako, Glostrup, Denmark) was added after washing and incubated. Subsequently, the membrane was incubated with anti-rabbit IgG antibody conjugate with IR DyLight 4xPEG (1:10,000 dilution, Thermo Scientific, Waltham, MA, USA), and IgE antibody binding was visualised using the Odyssey ^®^ CLx Imaging System and binding intensity analyses using Image Studio 5.2 (LI-COR Biosciences, Lincoln, NE, USA).

### 2.7. Amino Acid Sequencing Using Mass Spectrometry

To confirm the identity of the expressed and purified protein, mass spectrometry was performed using a method described previously [20]. The proteins were digested using a trypsin spin column (Sigma, St. Louis, MO, USA), prepared according to the manufacturer’s instructions. Using an LTQ Orbitrap Elite (Thermo Scientific, Waltham, MA, USA) with a nano ESI interface and Ultimate 3000 RSLC nano-HPLC, eluted peptides were analysed at Bio21 Institute (Parkville, Australia). The obtained spectra were identified using Mascot search engine (Matrix Science, Boston, MA, USA) with our in-house database of the oyster proteome (https://www.uniprot.org/proteomes/UP000005408 (accessed on 12 April 2021), and in addition the common Repository of Adventitious Proteins sequences (https://www.thegpm.org/crap/ (accessed on 15 April 2019).

### 2.8. Analysis of Secondary Protein Structure Using CD Spectrometry

To determine the alpha-helical confirmation of natural and recombinant TM, circular dichroism (CD) spectrometry was performed [21]. Natural and recombinant TM were purified in PBS (pH 7.2) and adjusted to 3 µM. CD spectroscopy was performed on a J715 Spectropolarimeter (Jasco, Oklahoma City, OK, USA) under continuous nitrogen flushing (25 °C). The analysis was performed over a wavelength range of 190–260 nm in a 10 mm quartz cuvette. TM samples were scanned in 0.2-nm steps with a bandwidth of 1 nm at 100 nm/min over eight scans. Results are expressed as mean residual ellipticity (θ) after subtracting the blank spectrum.

### 2.9. Evaluation of IgE-Reactivity Using ELISA

One microgram of TM in carbonate buffer pH 9.6 was added to each well of a 96-well EIA/RIA plate (Costar, St. Louis, MO, USA) and incubated overnight at 4 °C. The plate was washed four times using 0.05% Tween 20/PBS (PBS-T) and subsequently blocked using 1x Casein Blocking Buffer (Sigma-Aldrich) in PBST. After 1 h incubation, the plate was washed four times, and wells were incubated with 100 µL of serum diluted 1:10 in 0.2× casein/PBST at room temperature for 3 h while shaking. Rabbit anti-human IgE antibody (1:4000 dilution; Dako) and subsequently goat anti-rabbit IgG-HRP (1:1000 dilution; Promega) were added to wells and plates incubated at RT. After being washed with PBS, IgE antibody was detected using 3,3′,5,5′-Tetramethylbenzidine substrate (Invitrogen). After 5 min, the reaction was terminated using 1 M HCl and absorbance was measured at 450 nm by spectrophotometry (BMG LABTECH, Mornington, Australia).

## 3. Results

### 3.1. SDS-PAGE and IgE-Antibody Reactivity of Pacific Oyster Extracts

**Figure 1:**
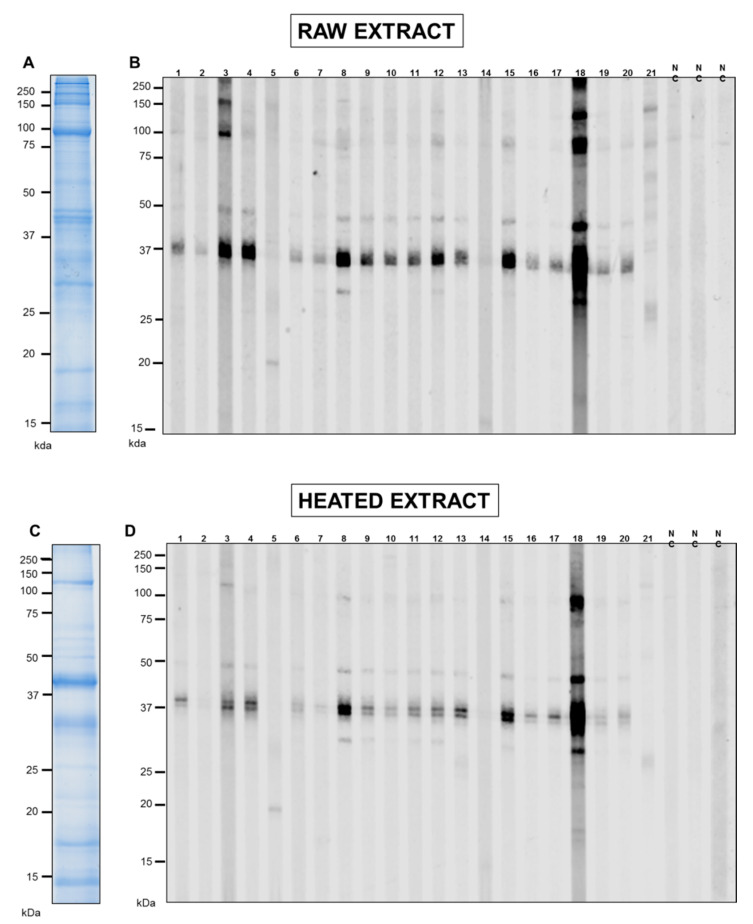
IgE reactivity of oyster-sensitised patients to raw and heated oyster extract (**top** and **bottom**). The extracts were separated by SDS-PAGE and stained with Coomassie Brilliant Blue (**A**,**C**). Immunoblots were performed with sera from 21 oyster-sensitised patients (1–21, **B**,**D**) as well as from 3 individuals non-allergic to oyster (NC).

The analysis of raw and cooked extracts of Pacific oyster by 1-dimensional SDS-PAGE showed various proteins with a molecular weight ranging from 10 to 200 kDa (Figure 1). Heat treatment reduced the number of proteins, particularly proteins with high molecular weight (Figure 1C), while heat-stable proteins were observed in the heated extract. In contrast, several protein bands that appeared in the heated extract were absent in the raw extract, demonstrating heat-induced modification of high molecular weight proteins by fragmentation or dimerization of certain proteins. TM, a heat-stable protein, was prominent in the heated extract at 38–40 kDa. These findings were consistent with the results of a previous study [6] analysing the protein profile of raw and heated extract using 2-dimensional SDS-PAGE and detailed mass spectrometry to identify IgE antibody-binding proteins. 

**Figure 2:**
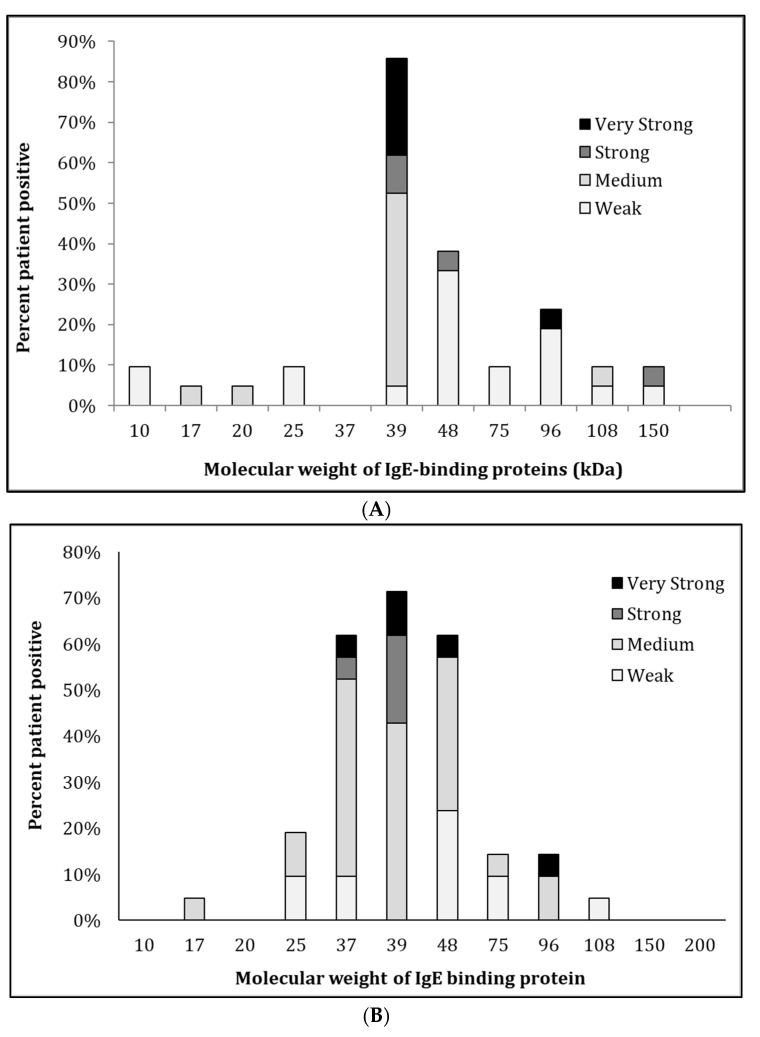
Allergogram analysis of IgE-binding patterns to proteins in the raw (**A**) and heated (**B**) oyster extracts. IgE-binding intensities were measured using Image Studio software and graded as weak, medium, strong and very strong. The percentage of patients reactivity for each IgE-binding intensity is shown.

IgE-reactivity of proteins in the oyster extracts was determined by immunoblotting using sera from 21 subjects with an elevated sIgE titre to whole oyster by Immuno-CAP (Figure 1B,C). The IgE-binding intensities are summarised in the allergrograms in Figure 2. IgE bands were marked as positive when their intensity was above the average of negative controls plus two standard deviations. In general, more IgE-reactive bands in the raw extract (Figure 1B) were observed than in heated extract (Figure 1D). However, this heated extract demonstrated an increased IgE binding intensity, particularly at 37 kDa. Moreover, 85% of the analysed individuals recognised proteins in the 38–40 kDa region in both extracts. Interestingly, those patients with IgE binding to TM also showed IgE binding to proteins at 48–50 kDa, although with intensity lower than that of TM. Other IgE binding bands most likely consisted of peptidyl-prolyl cis-trans isomerase (25 kDA), enolase (48 kDa), paramyosin (96 kDa), and myosin heavy chain (150 KDa) as previously identified [6]. However, the subsequent analysis focuses on the major IgE binding oyster allergen, TM, recognised by over 80% of sensitised individuals.

### 3.2. Sequencing and Characterisation of Pacific Oyster TM

**Figure 3:**
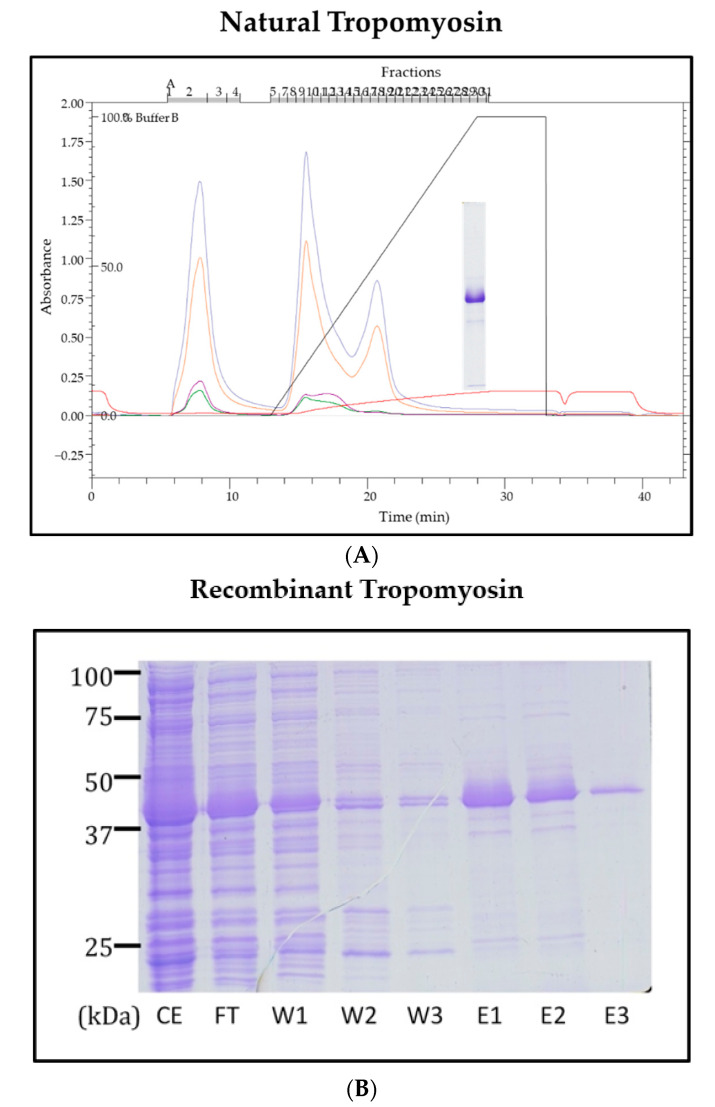
Purification profile of natural (**A**) and recombinant (**B**) tropomyosin of Pacific oyster. Natural tropomyosin was purified using CHT™ Ceramic Hydroxyapatite. The increase in the absorbance was measured at 280 nm (blue line) and 220 nm (red line) and 31 eluted fractions were collected and analysed by SDS-PAGE. The 17th, 18th, and 19th peak contained pure tropomyosin, appearing as a strong band at 39 kDa in the Coomassie-stained SDS-PAGE gel. Recombinant tropomyosin (3B) was purified using HisPur Ni-NTA with increasing concentration of imidazole. Purified tropomyosin was observed at 40 kDa due to six-His-tag fused to its N-terminal. Note for Figure 3B: CE = crude extract, FT = Flow through, W = Wash, E = Eluent.

To confirm the IgE-reactivity of TM, natural TM was purified from oyster extract using CHT™ Ceramic Hydroxyapatite ion-exchange chromatography (Figure 3A). Recombinant oyster TM was also expressed in an *E. coli* expression system and further purified using HisPur Ni-NTA Resin (Figure 3B). The cDNA sequence of the TM was published in GenBank under the accession number KY549366.1.

**Figure 4:**
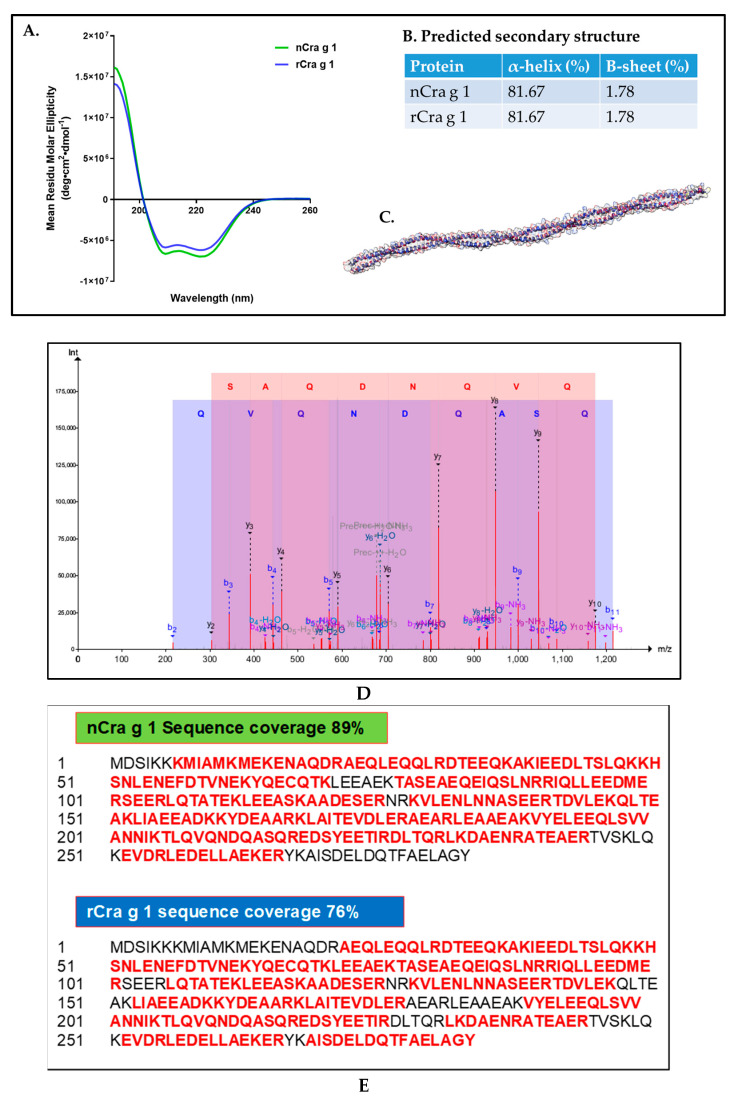
Structural analysis of the purified natural and recombinant tropomyosin. (**A**) Circular dichroism (CD) spectroscopy profile of natural (green line) and recombinant tropomyosin (blue line). (**B**) The estimated structure of tropomyosin predicted using K2D3 web server [22], with the majority of the structure consisting of an alpha-helix (**C**). (**D**) Representative product spectra of unique peptides of TM generated from trypsin digestion. (**E**) Peptide sequences identified by mass spectrometry are highlighted in red colour, overlaying the genomic deducted full-length sequence (black).

The structural properties of natural and recombinant TM were determined using CD-spectroscopy (Figure 4A) and LC-MS/MS mass spectrometry (Figure 4D). The CD spectra confirmed the identical secondary structure between natural and recombinant TMs, although recombinant TM possessed slightly higher minima of the mean residual ellipticity (MRE) at λ = 209 and 222 nm as predicted using K2D3 web servers [22]. Consistent with previous studies, the structure of TM is dominated by α-helical signal (81.67%) and confirmed by the 3D structure modelling of TM (Figure 4B,C) [20,23]. LC-MS/MS mass spectrometry was used to confirm that the amino acid sequences of the purified natural and recombinant proteins are indeed TM. Figure 4D displays a representatives of unique peptide belonging to TM and confirming that the purified proteins are TM. Moreover, the sequence coverage of TM is excellent, with the peptides identified by mass spectrometry covering 89% and 76% of the natural (nCra g 1) and recombinant TM (rCra g 1) amino acid sequence, respectively (Figure 4E). 

### 3.3. IgE-Recognition of Oyster-Sensitised Subjects to Natural and Recombinant TM

**Figure 5:**
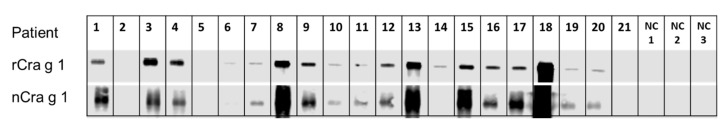
IgE reactivity analysis of natural and recombinant Pacific oyster TM using immunoblotting with 21 oyster-sensitised patients. rCra g 1 = purified recombinant Pacific oyster tropomyosin, nCra g 1 = purified natural Pacific oyster tropomyosin.

Eighty six percent of the 21 oyster-sensitised subjects showed IgE binding to both natural and recombinant TM (Figure 5). The intensity of IgE binding to natural and recombinant TM was comparable, except in subjects 6 and 14, which showed weak IgE binding to recombinant TM, but very weak binding to natural TM. 

### 3.4. Immunological IgE-Reactivity Analysis Using ELISA

Immunological reactivity between molluscs, crustaceans, and house dust mites is often reported, possibly due to the high amino acid sequence identity of their allergens. TM is known as invertebrate pan-allergen responsible for major IgE reactivity in oyster, prawn, and cockroach, while much lower immunological reactivity is seen to TM in mites [24].

**Figure 6:**
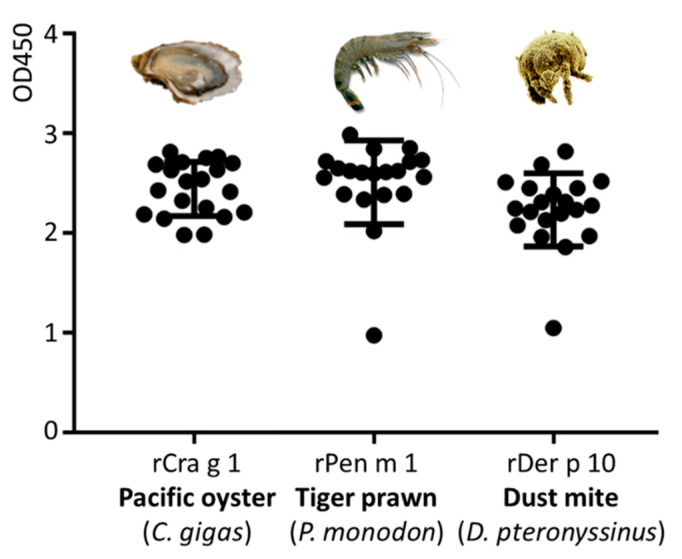
Patient serum IgE reactivity analysed by ELISA for three purified tropomyosins: Cra g 1 (Pacific oyster), Pen m 1 (Black tiger prawn) and Der p 10 (house dust mite) (*N* = 18).

In this study, the immunological reactivity to purified Pacific oyster TM (Cra g 1), black tiger prawn TM (Pen m 1), and house dust mite TM (Der p 10) in 18 TM-reactive subjects was analysed by ELISA (Figure 6). The IgE reacivity to Pen m 1 was markedly higher as compared to Der p 10 and Cra g 1. The majority of subjects were significantly more reactive to Pen m 1 than to Cra g 1 or to Der p 10 (*p* < 0.001), with one exception for subject 14, where the reactivity to Cra g 1 was much higher than to Pen m 1 or to Der p 10 (*p* < 0.001). 

### 3.5. Tropomyosin Amino Acid Sequence Comparison and IgE-Binding Epitope Prediction

**Figure 7:**
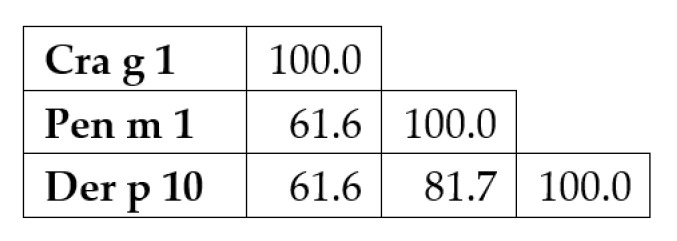
Percent identity matrix of tropomyosin sequences from Pacific oyster (Cra g 1), Black tiger prawn (Pen m 1) and House dust mite (Der p 10).

To understand the findings of the IgE-reactivity analysis of oyster, prawn, and dust mite TM by ELISA, a comparison of the amino acid sequence of TM from these three species was generated. The three TM sequences were aligned using MUSCLE (MUltiple Sequence Comparison by Log-Expectation) program in Mega 7 software with default parameter sets. Concurrent with the ELISA result of IgE reactivity where the majority of the subjects showed higher reactivity to Pen m 1 and Der p 10, the sequence alignment demonstrated that prawn TM is more closely related to dust mite TM rather than to oyster TM. Seventy-two and 126 amino acid substitutions were observed in Der p 10 and Cra g 1, respectively, compared to Pen m 1. Sequence identity between Pen m 1 and Der p 10 is 81.7%, while between Pen m 1 and Cra g 1 the identity is much less, 61.6% (Figure 7). 

**Figure 8:**
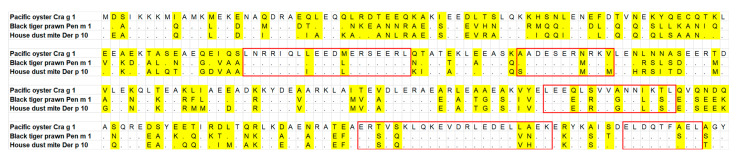
Amino acid sequence alignment of tropomyosin sequence from Pacific oyster (*Crassostrea gigas*), Black tiger prawn (*Penaeus monodon*) and house dust mite (*Dermatophagoides pteronyssinus*). Variable amino acids are shaded in yellow. The predicted IgE-binding epitopes responsible for cross-reactivity in those three species are identified by solid red boxes.

A previous study [25] showed that cross-reactivity occurs when the IgE antibody binds to proteins from other species having peptide with maximum two amino acid replacements as compared to the full length IgE-binding epitopes. Using this knowledge, five regions were identified between these three TMs, with two of these regions positioned near the C-terminal, most likely responsible for the demonstrated IgE binding cross-reactivity between oyster, prawn, and dust mite (Figure 8). 

## 4. Discussion

The development of accurate diagnostic tools for mollusc allergy is still facing difficulties due to the large diversity of edible mollusc species as well as the lack of purified natural or recombinant clinically relevant allergens. Clinical history is often unreliable as the patients cannot easily recall the exact mollusc species implicated in allergic reactions. While food challenges are regarded as the gold standard of food allergy diagnosis, their application has been associated with risks of severe reaction in the case of shellfish challenge due to high allergen potency and adult patient co-morbidities [26]. Several mollusc IgE-reactive proteins have been identified in previous studies [6,8]. However, they have not been fully characterised, limiting their application as diagnostic tools for mollusc allergy. In this study, the major allergen of the Pacific oyster was biochemically characterised, the IgE recognition in a large cohort of oyster allergic patients determined, and the IgE reactivity to purified recombinant generated TM from black tiger prawn (Pen m 1) and house dust mite (Der p 10) was compared. Subsequently, TM was registered with the WHO/IUIS as the first allergen from the Pacific oyster. 

In this study, 21 patients were analysed for their IgE reactivity against the raw and cooked extract of the Pacific oyster. Similarly to the findings of previous studies [6,8], more IgE-binding proteins were observed in the raw compared to the heated protein extract. However, the heated extract demonstrated higher IgE binding intensity than the raw extract for the same IgE-binding proteins. Pacific oyster is the most consumed species of mollusc [27]. While most of the shellfish species, such as prawns and crabs, are ingested after heat treatment, oysters are mostly consumed raw, thereby potentially presenting both heat-labile and heat-stable allergens to the consumer’s immune system. The results presented in this study are concurrent with the previous findings using a pool of patient sera but demonstrated diverse allergen recognition.

Different IgE-binding patterns of the 21 patients were observed, suggesting that different sensitisation profiles are present in oyster-allergic patients. The assessment also demonstrated that not all subjects showed IgE reactivity to TM. Although the majority of the patients recognised TM (>80%), three patients showed no reactivity to TM. Instead, these patients’ IgE recognised other proteins with molecular mass corresponding to those of other mollusc allergens, including peptidyl-prolyl cis-trans isomerase and paramyosin as also reported previously [24]. Additionally, over 50% of patients reacted to proteins at molecular weight ~50 kDa in the heated extract. The heating of shellfish protein extracts usually results in the loss of high molecular weight proteins. Mass spectrometry analysis of these IgE-binding bands revealed 15 different proteins with TM as the major component. Whether the IgE binding was due to TM isoforms, aggregates, or other proteins in the mixture is unclear. However, our recent investigations showed that TM from the Sydney rock oyster (*Saccostrea glomerata*) could be identified at 34, 39, 45, and 72 kDa in the heated extract, possibly due to degradation and aggregation during heat treatment [9].

The generation of purified recombinant allergens is essential for improved allergy diagnosis. Traditionally, the diagnosis of an allergy is performed using the extracts obtained from different allergen sources. These extracts, however, contain a mixture of non-allergenic and allergenic proteins and are often difficult to standardise the allergen content in these tests [28]. Current in vitro diagnostics are increasingly conducted at the molecular level, collectively referred to as component-resolved diagnosis [5,24]. Allergen purification is also an essential criterion for registration with the WHO/IUIS Allergen Nomenclature Sub-Committee. The current study reports for the first time the cloning, full sequence analysis and recombinant expression of the Pacific oyster TM. The recombinant TM shares 97% amino acid sequence similarity with previous published Pacific oyster TM, BAH10152.1. Mass spectrometry and CD spectroscopy analysis of purified natural and recombinant TM demonstrated both TMs have near identical properties. The immunological characteristic of the recombinant TM is also very similar to that of natural TM. Based on this study, the Pacific oyster TM has been registered and designated Cra g 1.0101 by the WHO/IUIS allergen sub-Committee (http://allergen.org/viewallergen.php?aid=902 (accessed on 12 December 2021) as a new oyster allergen. 

Molluscs, together with crustaceans, are termed as shellfish in fisheries terminology. However, taxonomically, those two groups belong to different families. Due to this assumption, crustacean-allergic patients are often advised to also avoid mollusc. Although allergen cross-reactivity between crustaceans and mollusc has been documented clinically and experimentally [29], group-specific allergy has also been reported [30], suggesting that the clinical recommendation of total seafood avoidance may not be accurate. In this study, IgE reactivity between oyster TM Cra g 1, prawn Pen m 1, and dust mite Der p 10 was evaluated by ELISA. The results showed that patients which recognised oyster TM also demonstrated IgE reactivity with Pen m 1 from prawn and Der p 10 from dust mite. All patients but one demonstrated higher IgE reactivity to Pen m 1 than to Cra g 1, in agreement with the ImmunoCAP results. TM is a pan-allergen and has been shown to be responsible for clinical and immunological cross-reactivity across different invertebrate species [31]. However, the IgE reactivity patterns did not correlate with the degree of amino acid sequence identity as the IgE reactivity of Cra g 1 was higher as compared to Der p 10. The identity between Pen m 1 and Der p 10 (81.7%) is considerably higher than between Pen m 1 and Cra g 1 (61.6%). Furthermore, the amino acid sequence identities of the predicted IgE-binding epitopes in Pen m 1 showed Cra g 1 to have more sequence mismatches compared to that of Der p 10, particularly at the N-terminal and middle section of the TM protein [32]. The contradiction between the ELISA results and the degree of amino acid sequence identity suggests that symmetrical cross-reactivity occurs in the studied patient cohort. 

## 5. Conclusions 

This is the first study on the identification of allergens in the Pacific oyster using a large cohort of oyster-sensitised patients. TM was confirmed as the major allergen and registered with the WHO/IUIS as Cra g 1, reacting with 18 out of 21 sensitised subjects analysed. Other observed IgE-binding proteins include previously identified allergens from other mollusc species. Patients with reactivity to oyster TM also demonstrated IgE reactivity to prawn and dust mite TM, although the degree of reactivity varied among patients. In summary, the findings of this study provide novel recombinant oyster allergens for the development of reliable component-resolved diagnostic assays for mollusc allergy and enable accurate dietary advice and guidance for future immunotherapeutic approaches. 

## Figures and Tables

**Table 1:** Characteristics of shellfish-allergic patients recruited for this study.

Patient ID	Specific IgE (kU_A_ L^–1^)			Implicated Species	Symptoms
	Oyster	Prawn	HDM		
1	0.93	10.10	8.73	Prawn	As, R, U
2	0.92	9.50	14.10	Prawn, crab, flounder	A, An, R
3	2.04	9.03	13.60	Calamari, snapper, tuna	R, An
4	3.75	9.82	2.66	Scallops, oyster, shellfish	An, U, pO
5	4.29	0.20	9.49	Sea perch, flake, rockling	An, pO
6	1.04	6.84	31.70	Mussel, scallops	An
7	0.49	2.57	1.99	Calamari, octopus	An
8	5.99	32.40	6.47	Shellfish	As, R, U, An
9	2.41	8.98	2.36	Shellfish	As, R, U, An
10	1.11	3.63	5.03	Crustaceans/molluscs	R, H, A
11	1.19	4.30	57.5	Prawn, crab meat and marinara mix	As
12	6.68	17.2	13.50	Salmon, crab, lobster, prawn	An
13	2.59	9.81	16.80	Prawns, calamari, fish	An
14	0.65	3.75	33.80	Oyster	GI
15	5.47	21.60	10.7	Calamari	U, As
16	1.35	5.42	40.20	Mollusc	An, A, U
17	1.08	6.73	6.90	Shellfish	U, A
18	35.8	>100	22.00	Shellfish	U, As
19	0.45	9.74	1.29	Shellfish	As
20	1.08	5.05	1.97	Pipis, squid	pO
21	7.32	2.84	1.95	Oyster	NI

NI, no information; A, anaphylaxis; An, angioedema; As, asthma; H, hypotension; R, rhinitis; U, urticaria; pO, periorbital edema.

## Data Availability

Not applicable.
